# Cross-domain transfer learning from peptides to metabolites using a multi-property fine-tuned LLM

**DOI:** 10.1093/bioinformatics/btag493

**Published:** 2026-07-03

**Authors:** Uchenna Alex Anyaegbunam, David Teschner, Thierry Schmidlin, Andreas Hildebrandt, Johannes U Mayer, Maximilian Sprang, Miguel A Andrade-Navarro

**Affiliations:** Computational Biology and Data Mining group (CBDM), Institute of Organismic and Molecular Evolution (iOME), Johannes Gutenberg University, Mainz, 55128, Germany; Institute of Computer Science, Johannes-Gutenberg University, Mainz, 55128, Germany; Institute for Quantitative and Computational Biosciences (IQCB), Johannes-Gutenberg University, Mainz, 55128, Germany; Institute of Immunology, University Medical Center of the Johannes-Gutenberg University Mainz, Mainz, 55131, Germany; Research Centre for Immunotherapy (FZI), University Medical Center of the Johannes-Gutenberg University Mainz, Mainz, 55131, Germany; Institute of Computer Science, Johannes-Gutenberg University, Mainz, 55128, Germany; Institute for Quantitative and Computational Biosciences (IQCB), Johannes-Gutenberg University, Mainz, 55128, Germany; Institute for Quantitative and Computational Biosciences (IQCB), Johannes-Gutenberg University, Mainz, 55128, Germany; Research Centre for Immunotherapy (FZI), University Medical Center of the Johannes-Gutenberg University Mainz, Mainz, 55131, Germany; Department of Dermatology, University Medical Center of the Johannes Gutenberg University Mainz, Mainz, 55131, Germany; Computational Biology and Data Mining group (CBDM), Institute of Organismic and Molecular Evolution (iOME), Johannes Gutenberg University, Mainz, 55128, Germany; Institute for Quantitative and Computational Biosciences (IQCB), Johannes-Gutenberg University, Mainz, 55128, Germany; Department of Dermatology, University Medical Center of the Johannes Gutenberg University Mainz, Mainz, 55131, Germany; Computational Biology and Data Mining group (CBDM), Institute of Organismic and Molecular Evolution (iOME), Johannes Gutenberg University, Mainz, 55128, Germany

## Abstract

**Motivation:**

Accurate liquid chromatography retention time (RT) prediction is a critical component of compound identification in metabolomics and lipidomics. However, existing RT prediction approaches are often limited by the scarcity of experimental RT measurements for many molecular classes, restricting model generalization and the construction of comprehensive RT libraries. Transfer learning from data-rich chemical domains offers a potential strategy to overcome these limitations, but its effectiveness for metabolite RT prediction remains insufficiently explored.

**Results:**

We developed a transfer learning framework based on ChemBERTa that leverages large peptide datasets to improve metabolite RT prediction under data-sparse conditions. A peptide-pretrained model was trained using a multi-task objective that jointly predicted RT and seven RDKit-derived molecular descriptors. Compared with an RT-only model, the multi-task approach learned more robust chemical representations and demonstrated superior generalization to metabolites, achieving a median test R² of 0.842 versus 0.820. When transferred to metabolite RT prediction, the multi-task pretrained model substantially outperformed models trained from scratch at low-data regimes. Using only 3% of metabolite training data (2129 compounds), transfer learning achieved a median test R² of 0.322 compared with 0.216 for the baseline model, while reducing MAE from 131.7 to 114.9. Significant improvements were also observed at 5% and 10% training fractions, with benefits gradually diminishing as larger metabolite datasets became available. In contrast, a peptide-pretrained single-task RT model showed performance comparable to the baseline, indicating that the observed gains arise primarily from multi-task molecular property learning rather than peptide pretraining alone. These findings demonstrate that multi-task transfer learning provides an effective and scalable strategy for improving RT prediction in metabolomics, particularly when experimental training data are limited.

**Availability:**

Freely available on https://github.com/uchealex/CHEMBEDDING.

## 1 Introduction

Accurately predicting molecular properties from chemical structure is fundamental to computational bioinformatics and drives advancements in fields such as drug discovery (Lavecchia 2015), metabolomics (Wishart 2019), and systems biology. Among these properties, liquid chromatography retention time (RT) is a major analytical metric. RT is essential for separating, identifying, and characterizing complex mixtures of compounds, such as metabolites and lipids, in biological samples (Stanstrup *et al.* 2015, Bros *et al.* 2023). Computational prediction of RT can dramatically accelerate the annotation of unknowns in untargeted omics studies, which remains a major bottleneck (Domingo-Almenara *et al.* 2019). This challenge is particularly observed in lipidomics and metabolomics, where chemical diversity is high, but experimentally measured RTs are available for only a small fraction of known molecules, and might differ substantially depending on which chromatographic methodology is employed. However, a persistent limitation is the scarcity of large, high-quality, labeled experimental datasets for many molecular classes (Ching *et al.* 2018, Yang *et al.* 2019).

Transfer learning (TL) has emerged as a powerful strategy to overcome data scarcity (Pan and Yang 2010, Weiss *et al.* 2016). Knowledge gained from a data-rich source task [as in bottom-up proteomics, where predicting peptide properties such as retention time, ion-mobility, or fragmentation patterns are well established, and rich datasets exist to train predictors for those tasks (Zeng *et al.* 2022, Buur *et al.* 2024)] is transferred to improve learning on a data-poor target task (such as predicting these properties for lipids and metabolites) (Yosinski *et al.* 2014, Zhuang *et al.* 2021). The underlying hypothesis is that neural networks can learn fundamental, domain-independent chemical principles: relationships between structure, polarity, lipophilicity, size, etc., during initial training, and these learned representations can be transferred across related biochemical domains (Xu *et al.* 2017, Ramsundar *et al.* 2019). Recent work in proteomics has provided strong empirical support for this hypothesis. For example, the DeepLC framework demonstrated that peptide RT prediction models pre-trained on large datasets can be efficiently adapted via transfer learning to new chromatographic conditions and even to peptides bearing chemically distinct modifications, without retraining from scratch (Bouwmeester *et al.* 2025). These results established transfer learning as a robust mechanism for adapting chromatographic predictors under distributional shift. However, systematic investigation of cross-domain transfer, particularly from peptides to chemically distinct molecular classes such as metabolites, remains limited.

At the same time, large language models (LLMs) have revolutionized representation learning in chemistry. Models like ChemBERTa are pre-trained on a vast amount of SMILES strings, treating chemical structures as a language (Wang *et al.* 2019, Chithrananda *et al.* 2020). Self-supervised pre-training allows the model to learn rich, contextual embeddings of atoms and functional groups, effectively capturing the ‘syntax and semantics’ of chemistry without labeled data (Devlin *et al.* 2019, Rives *et al.* 2021). Such models serve as versatile ‘foundation models’ that can be fine-tuned to specific downstream tasks with relatively little data (Bommasani *et al.* 2021, Zvyagin *et al.* 2022). In metabolomics, transformer-based architectures pre-trained on large molecular corpora have already demonstrated strong performance in RT prediction and transfer across chromatographic systems (Xue *et al.* 2024, Mazraedoost *et al.* 2025).

Simply fine-tuning a base chemical LLM on a single target task (e.g. RT prediction) can be effective, but in extremely data-sparse scenarios it remains prone to overfitting and may fail to learn the most robust, generalizable features (Chen *et al.* 2021). Multi-task learning (MTL) provides a compelling solution by training one model on multiple related tasks simultaneously (Caruana 1997, Zhang and Yang 2022). In molecular property prediction, augmenting the primary task (RT) with auxiliary tasks (such as predicting fundamental physicochemical descriptors) imposes a strong inductive bias (Wang *et al.* 2023, Gan *et al.* 2024). The model’s internal representation, in turn, then encodes a better understanding of chemistry. For example, jointly predicting RDKit descriptors (Landrum 2020) like molecular weight, polar surface area, hydrogen-bond donors/acceptors, rotatable bonds, and aromatic ring count, pushes the model towards learning structural principles that govern both chromatographic behavior and core molecular properties. The resulting learned features tend to be more robust and transferable than those learned from a single task alone. This strategy aligns with recent trends in metabolomics RT prediction, where combining structural representations with auxiliary molecular information has been shown to improve transferability across experimental conditions (Xue *et al.* 2024).

Notably, peptides and metabolites/lipids, despite differences in polymeric versus modular structures, are governed by shared physicochemical principles (e.g. hydrophobicity, charge, volume) that dictate their separation in reversed-phase liquid chromatography (Cajka and Fiehn 2016, Kyle 2017). A model that learns to predict peptide RT alongside its fundamental descriptors may therefore develop feature representations that are directly relevant to metabolites. This strategy aligns with the vision of building ‘foundation models for science’ that bridge data-rich and data-poor domains (Greener *et al.* 2022, Xu *et al.* 2022). Despite this potential, there has been little systematic investigation of whether multi-property pre-training on peptide data yields features that transfer effectively to metabolite RT prediction. In this study, we propose and evaluate a novel pipeline for metabolite RT prediction under data-limited conditions. Our approach is founded on two main hypotheses: (1) A ChemBERTa LLM fine-tuned with a multi-task objective on peptide data (predicting both RT and seven RDKit-derived descriptors) will learn chemical representations that are superior and more transferable than those from a model fine-tuned on peptide RT alone. (2) These enriched representations will enable cross-domain transfer to metabolite RT prediction, with benefits that are most pronounced in low-data domains.

To test these hypotheses, we first develop a multi-property model (ChemBERTa+RDKit) and a single-task baseline model (ChemBERTa-RT) using a large public peptide dataset. We demonstrate that the multi-property model achieves better generalization on both peptide and metabolite RT prediction compared to the single-task model. We then use the multi-property peptide model as a pre-trained source for transfer learning to metabolites, systematically fine-tuning it on subsets of metabolite RT data ranging from 3% to 100%. We compare its performance to baseline models trained from scratch on the same metabolite subsets. Our results provide clear, practical guidelines for deploying transfer learning in resource-constrained metabolomics and lipidomics: we observe measurable gains when metabolite data is very limited relative to the peptide data (about 1–4% of the peptide pre-training data), and parity of performance thereafter (about 5–25% of the peptide pre-training data). Overall, this multi-task transfer approach leverages large public peptide datasets to solve small-scale data challenges in omics.

## 2 Methods

### 2.1 Datasets and preprocessing

Peptide retention time data: We used a large-scale peptide RT dataset of ∼250 000 entries from the ProteomeTools project (Zolg *et al.* 2017). The processed data was accessed via the ProteomeTools repository on ProteomicsML (Rehfeldt *et al.* 2023), which provides standardized training/test splits for machine learning. Each example includes a SMILES string for a unique peptide and an experimental LC-MS/MS retention time.

**Figure 1:**
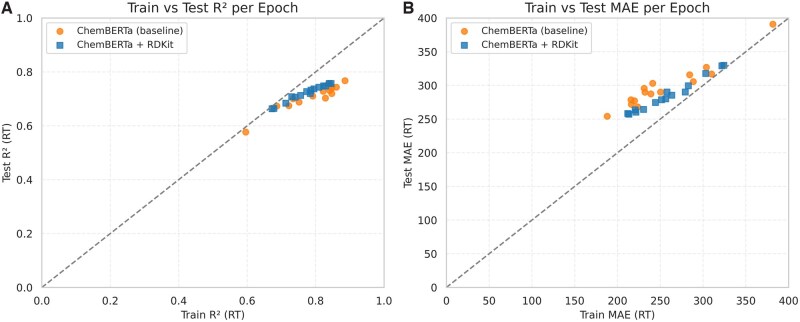
Multi-property training improves peptide retention time prediction. (A) Epoch-wise scatter plot of test versus train R^2^. The ChemBERTa+RDKit model (blue) shows a consistent upward shift in test R^2^ compared to the ChemBERTa baseline (orange), reflecting enhanced predictive performance on independent peptide data. (B) Epoch-wise scatter plot of test versus train MAE. The ChemBERTa+RDKit model (blue) achieves lower test MAE with reduced epoch-to-epoch variability, confirming that joint training with molecular descriptors yields a more reliable and generalizable model for peptide retention time.

**Figure 2:**
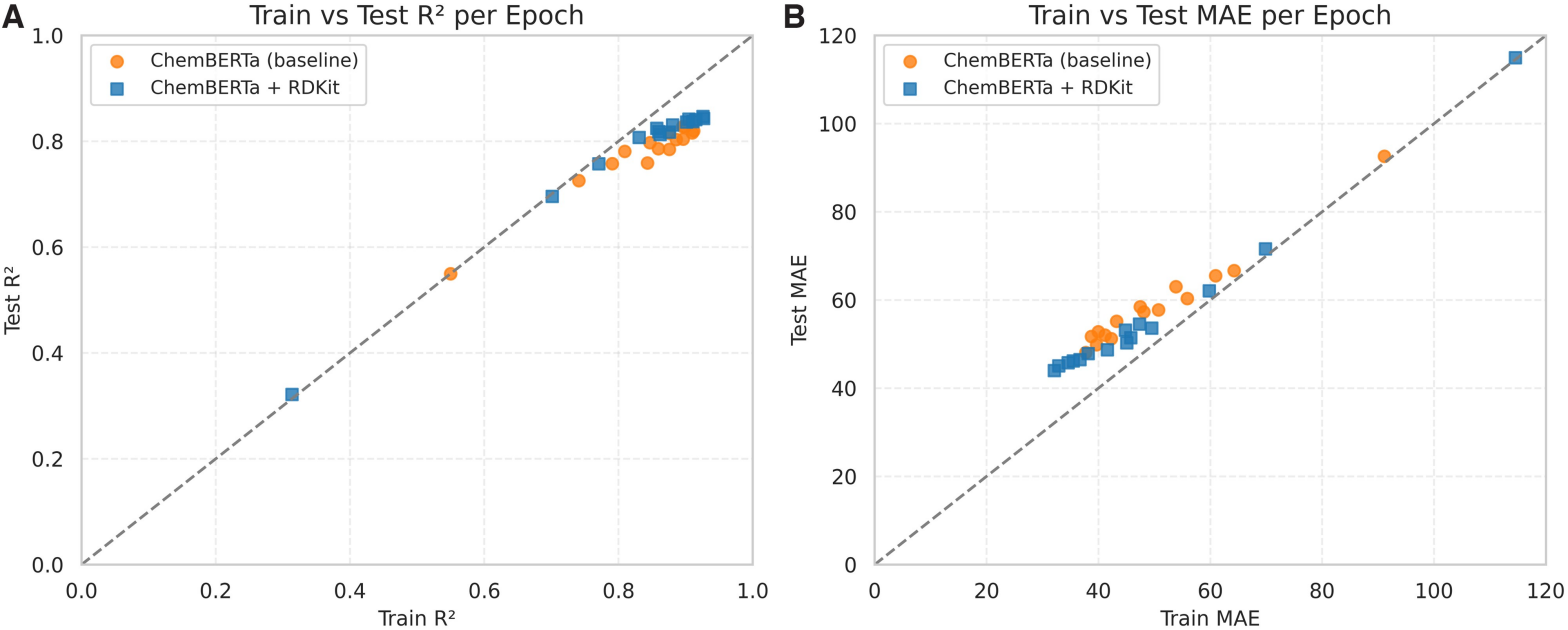
Multi-property training improves metabolite retention time prediction. (A) Scatter plot of test versus train R^2^ for each of the 15 training epochs. Each point represents model performance at one epoch. The ChemBERTa+RDKit model (blue) achieves higher test R^2^ values for comparable train R^2^ values than the ChemBERTa baseline (orange), indicating better generalization. (B) Scatter plot of test versus train MAE (Mean Absolute Error, in seconds) for each epoch. Points for the ChemBERTa+RDKit model (blue) are clustered toward lower test MAE values, demonstrating more accurate and stable predictions on unseen metabolite data compared to the baseline (orange). The multi-property model leverages RDKit-derived descriptors (MW, TPSA, HBD, HBA, etc.) as auxiliary tasks to learn more robust molecular representation.

Metabolite retention time data: For the target task, we used a metabolite RT dataset derived from the METLIN SMRT database (Domingo-Almenara *et al.* 2019). This dataset contains LC-MS retention times for diverse metabolite molecules. We extracted canonical SMILES and RT values, and we constructed standardized splits (training and test) with 63 163 and 7798 molecules, respectively, as used by Mazraedoost *et al.* (2025) for only Subsection 3.1, Figs. 1 and 2. To simulate data-sparse scenarios, the metabolite data set was randomly subsampled from the full dataset (70 961 molecules) for each fold in the 5-fold cross-validation at fractions of 3%, 5%, 10%, 25%, and 50% (corresponding to 2129; 3548; 7096; 17 740 and 35 480 metabolites, respectively). Molecular descriptors: For both peptide and metabolite molecules, we computed seven fundamental physicochemical descriptors from the SMILES using the RDKit cheminformatics toolkit (Landrum 2020). The descriptors were molecular weight (MW), topological polar surface area (TPSA), number of hydrogen bond donors (HBD), number of hydrogen bond acceptors (HBA), rotatable bond count, aromatic ring count, and heavy atom count. These descriptors served as auxiliary targets in the multi-task learning framework.

Data scaling: All target variables (RT and the seven descriptors) were normalized to [0, 1] using MinMaxScaler from scikit-learn (Pedregosa *et al.* 2011), which was fit on the training data. This normalization is particularly important in chromatography, as absolute RT values strongly depend on experimental conditions such as gradient length, which can range from a few minutes to over two hours across studies. In the transfer learning experiments, the metabolite RT values were scaled using the parameters learned from the peptide RT training data. This alignment ensured that the metabolite RT distribution matched the pre-trained model’s output scale, which is critical for effective transfer.

### 2.2 Model architecture and pre-training

We based our models on the ChemBERTa-zinc-base-v1 transformer encoder (Chithrananda *et al.* 2020), which is a variant of RoBERTa (Liu *et al.* 2019) pre-trained on the ZINC15 database (Sterling and Irwin 2015). This model has 12 transformer layers, a hidden embedding size of 768, and ∼86M parameters. The special CLS token’s output from the final layer serves as the pooled molecular embedding. All ChemBERTa models used were fine-tuned starting with the default weights.

We implemented two model variants:

1. Single-Task Baseline (ChemBERTa-RT): The pre-trained ChemBERTa encoder feeds into a single linear output node for RT prediction only.
2. Multi-Property (ChemBERTa+RDKit): The ChemBERTa encoder feeds into a linear layer producing an 8-dimensional output (one for RT plus one for each of the seven RDKit descriptors). This model is trained to jointly regress RT and descriptors in a multi-task setup.

For the multi-property model, the loss is the sum of mean squared errors (MSE) across all eight outputs. The code for model definition and training is available at our public GitHub repository (github.com/uchealex/chembedding). In all cases, we used the CLS token embedding as input to the regression head, since this token’s representation aggregates information from the entire SMILES sequence via self-attention.

### 2.3 Training procedures

Multi-task pre-training on peptides: We first trained the ChemBERTa+RDKit model on the full peptide dataset. Training was run for 15 epochs, minimizing the summed MSE loss across the eight outputs. We used the AdamW optimizer (Loshchilov and Hutter 2019) with a learning rate of 2.5 × 10–5, batch size of 16, and standard linear warmup/decay schedules. For comparison, we also trained a single-task ChemBERTa-RT model (same hyperparameters but only one output).

Transfer learning to metabolites: We used the trained multi-property peptide model as the source for metabolite RT prediction. Training was done for 10 epochs under 5-fold cross-validation. Specifically, we created a new target model by copying the transformer encoder weights from the peptide model and randomly reinitializing a fresh regression head (single output for RT). This model was then fine-tuned on each metabolite subset (3%, 5%, 10%, 25%, 50%, 100% of data). Each metabolite percentage subset was randomly sampled from the full data at each fold, to get a 5-fold cross-validation for model types. For 100% of the dataset, the standard 5-fold cross-validation from scikit-learn was applied on the whole dataset. For all percentage sub-samples, the train-test split was done using scikit-learn function.

We used the same hyperparameters as above (AdamW, lr = 2.5e-5, 10 epochs, batch = 16, warmup). During fine-tuning, we updated all encoder weights to allow the learned peptide features to adapt to the metabolite domain.

For comparison, we also trained baseline models on each metabolite subset from scratch (randomly initialized ChemBERTa-zinc-base-v1, single RT output). These baselines used the same architecture and hyperparameters but no peptide pre-training, to measure performance without transfer. Finally, to check the effect of the RDKit properties on the transfer learning efficacy, we repeated the transfer learning procedure done above (for multitask peptide-pretrained model) using singletask peptide-pretrained model (that is, ChemBERTa pretrained on only the retention time of peptide).

### 2.4 Evaluation metrics and statistical analysis

We evaluated model performance on the held-out metabolite test set using the coefficient of determination (R^2^) and Mean Absolute Error (MAE). All evaluation metrics were computed after transforming model predictions and targets from the normalized [0,1] space back to their original, raw RT scale, ensuring that reported errors reflect experimentally meaningful units. For the multi-task peptide model, we report R^2^ and MAE for each of the eight outputs. For all metabolite RT models (transfer and baseline), we report R^2^ and MAE for RT only. Each model was trained for 15 epochs for Subsections 3.1, and 10 epochs for Subsections 3.2 and 3.3. At the end of each epoch, we recorded R^2^ and MAE on the test set. To robustly summarize performance and stability, we report the median, third quartile and interquartile range (IQR) (25th–75th percentile) of the epoch-wise metrics in Subsection 3.1, and 5-fold cross-validation metrics in Subsections 3.2 and 3.3 (Bouthillier *et al.* 2021). As recommended by Bouthillier *et al.* reporting median and IQR provides a clear measure of central tendency and variability of the training outcomes.

All models were implemented in PyTorch (Paszke *et al.* 2019) (v2.0) and the HuggingFace Transformers library (Wolf *et al.* 2020) (v4.30). Training was conducted on NVIDIA Tesla GPUs (T4 or V100) via Google Colab. Data processing and analysis used pandas and scikit-learn (Pedregosa *et al.* 2011).

## 3 Results

### 3.1 Multi-property training with RDKit descriptors enhances peptide and metabolite model generalization

We first examined whether joint training on RT and seven computed descriptors would improve the ChemBERTa model’s predictive power. The multi-property model (ChemBERTa+RDKit) was trained to predict RT and the seven RDKit descriptors (MW, TPSA, HBD, HBA, rotatable bonds, aromatic rings, heavy atoms computed from SMILES). We compared this to the single-task baseline trained on RT only.

We evaluated each model configuration across 15 training epochs. Reported metrics are the median and IQR of epoch-wise R^2^ and MAE, providing a robust measure of performance and stability. On peptide RT prediction, the multi-property model showed markedly improved generalization and stability (Fig. 1). It achieved a final test R^2^ of 0.757 (IQR: 0.737–0.757) versus 0.743 (IQR: 0.719–0.767) for the baseline. The final test MAE was substantially lower (258.3 vs. 295.6, both in same normalized units). Figure 1’s epoch-wise scatter shows the multi-property model’s R^2^ values are consistently higher, and its MAE values are consistently lower and less variable. This indicates that auxiliary descriptor prediction acts as a regularizer, guiding the model to learn a more robust, generalizable representation of peptide chemistry.

We observed a similar advantage on metabolite RT prediction (Fig. 2). The ChemBERTa+RDKit model attained a higher median test R^2^ (0.842, IQR: 0.817–0.847) than the baseline (0.820, IQR: 0.786–0.819), and a lower median test MAE (45.07 vs. 52.78). Its epoch-wise results cluster at higher R^2^ and lower MAE with tighter spread. The consistent improvement across both domains strongly supports the MTL paradigm. Jointly predicting auxiliary descriptors provides a rich inductive bias, forcing the model to build a comprehensive, physically grounded molecular representation. The model thereby learns underlying structural principles rather than memorizing dataset-specific correlations.

To probe whether RT is conditionable on the coarse RDKit descriptors, we trained predictors of increasing complexity (Ridge, Gradient Boosting and Random Forest on the seven RDKit features, together with linear heads on the frozen ChemBERTa CLS embedding with and without RDKit concatenation) under 5-fold cross-validation on the pooled metabolite dataset (Fig. S1). All baselines extracted a non-trivial RT signal from the descriptors alone (up to R^2^ ≈ 0.54 for Random Forest), confirming that the RDKit features carry a learnable signal that, once exposed to ChemBERTa as auxiliary multi-task targets, gives the encoder a stronger low-data conditioning signal and helps explain why the fine-tuned ChemBERTa+RDKit model significantly surpasses the single-task baseline (R^2^ = 0.842 vs. 0.820).

In summary, augmenting RT prediction with fundamental descriptors significantly improves model accuracy and robustness for both peptides and metabolites.

#### 3.2.1 Transfer learning at 3%, 5% and 10% metabolite data: consistent gains from multi-task pretraining

**Figure 3:**
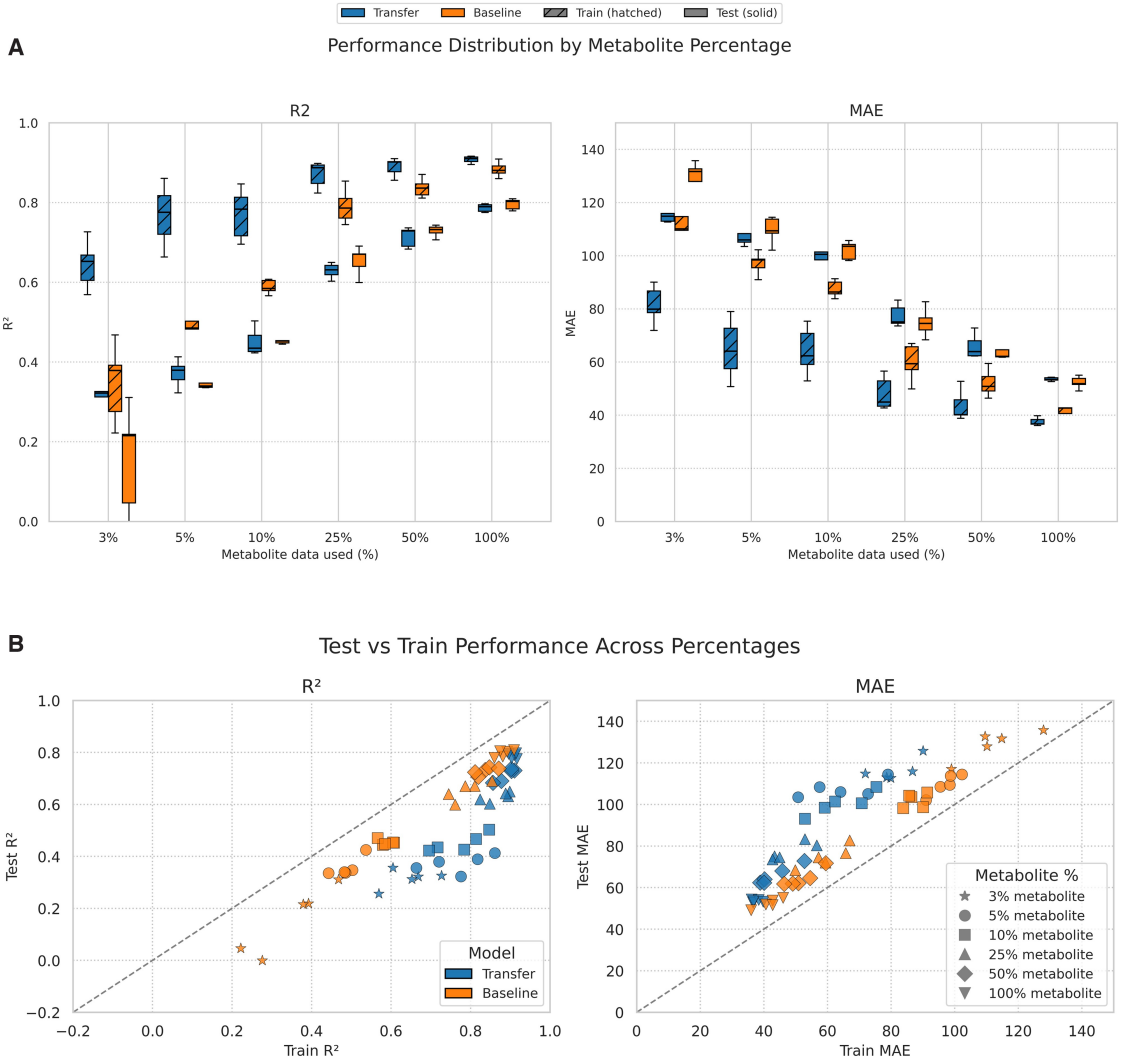
Transfer learning performance across 5-folds cross-validation for varying metabolite data amounts. (A) Boxplots of test R^2^ (median and IQR) across 5-fold cross-validation. At 3 to 5% data, Transfer (blue) shows higher median R^2^ and third quartile (Q3) relative to Baseline (orange). At 10% data, Transfer has a slightly lower median R^2^ but a higher third quartile (Q3) and better MAE. From 25% to 100% data, the baseline achieves equal or slightly higher median R^2^, with overlapping IQRs. This pattern indicates that transfer benefits are most pronounced only under extreme data scarcity. Boxplots of test MAE (median and IQR): Transfer learning yields lower median MAE at 3 to 10% data, with narrower IQRs indicating more stable predictions. At 25–50% data, MAE values are similar, though the baseline shows a slight edge at 50%. At 100% data, the baseline achieves a slightly lower median MAE, confirming that with abundant metabolite data, training from scratch is at least as effective as transfer learning.

We evaluated transfer learning for metabolite retention time (RT) prediction under extreme data scarcity, using only 3% (2129 metabolites), 5% (3548 metabolites) and 10% (7096 metabolites) of the metabolite dataset. A ChemBERTa+RDKit model pretrained on a multi-task peptide task (retention time together with multiple RDKit-derived molecular properties) was fine-tuned on these subsets and compared to baseline models trained from scratch. Performance was assessed by 5-fold cross-validation; results are reported as median and third quartile (Q3) test R^2^ and MAE across the five replicates (Fig. 3, Table S3).

At 3% of metabolite data, transfer learning clearly outperformed the baseline. The transferred model achieved a median test R^2^ of 0.322 (Q3 = 0.326), substantially higher than the baseline’s median of 0.216 (Q3 = 0.219). The transferred model also showed a much lower median MAE (114.9, Q3 = 115.9) compared to the baseline (131.7, Q3 = 132.7). Thus, even with only 3% of the data, the multi-task pretrained model provides a strong advantage.

At 5% of metabolite data, the advantage persisted. The transferred model achieved a median test R^2^ of 0.380 (Q3 = 0.389), versus the baseline’s median of 0.340 (Q3 = 0.347). The transferred model also showed a lower median MAE (106.0, Q3 = 108.3) compared to the baseline (109.4, Q3 = 113.8). The third quartile values indicate that three-quarters of transfer runs exceeded an R^2^ of 0.356 and an MAE below 108.3, whereas the baseline’s upper quartile only reached 0.347 for R^2^ and exceeded 113.8 for MAE. Hence, at 5% data, transfer learning is superior in both central tendency and upper-range performance.

At 10% of metabolite data, the pattern became more nuanced. The median test R^2^ for transfer learning was 0.435 (Q3 = 0.467), which was slightly lower than the baseline’s median of 0.453 (Q3 = 0.454). However, the third quartile (Q3) of R^2^ was higher for transfer learning (0.467 vs. 0.454), meaning that the best 25% of transfer runs achieved a higher R^2^ than the best 25% of baseline runs. For MAE, transfer learning was superior across both median (100.6 vs. 103.6) and Q3 (101.4 vs. 104.2). Thus, while a typical transfer run explained slightly less variance than a typical baseline run, the top-quartile transfer runs were clearly better in both R^2^ and MAE, indicating a higher performance ceiling.

Shading indicates train/test split.

(B) Test R^2^ across 5-fold cross-validation for models trained on 3%, 5%, 10%, 25%, 50%, and 100% of the metabolite dataset. The peptide-pretrained (Transfer) model (blue) outperforms the baseline (orange) at 3% and 5% data. At 10% data, Transfer shows a slightly lower median R^2^ but a higher third quartile (see Fig. 3B). At 25–100% data, the baseline matches or slightly exceeds Transfer in median R^2^.

Test MAE across 5-fold cross-validation for the same conditions: The Transfer model consistently achieves lower MAE than the baseline at 5% and 10% data, converging faster and maintaining a lower error floor. At 25–100% data, MAE values are similar between models, though the baseline shows a slight advantage at 50% and 100%.

#### 3.2.2 Role of auxiliary RDKit properties: single-task peptide-pretrained model yields approximate parity with baseline model

**Figure 4:**
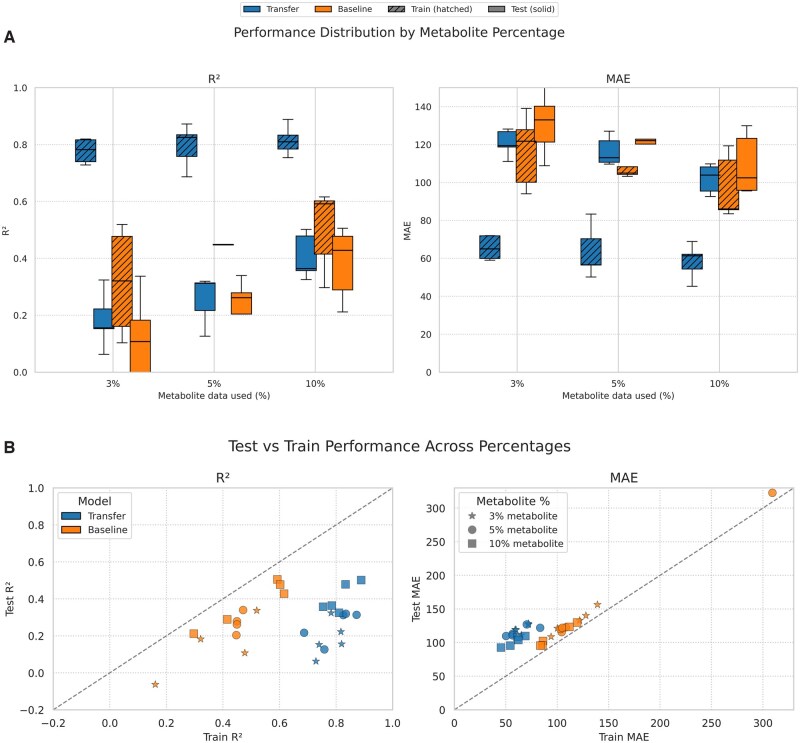
Comparison between single-task peptide-pretrained model and baseline model shows little to no transfer learning gains. (A) Boxplot comparison of test set R^2^ and MAE at 3%, 5% and 10% metabolite data. For each data fraction, five-fold cross-validation results are shown for the single-task pretrained (Transfer) model versus the baseline model trained from scratch. At all three sampling rates, the distributions of test R^2^ for the transfer model overlap substantially with those of the baseline, with no consistent shift toward higher R^2^. Similarly, MAE distributions are nearly superimposable. The median test R^2^ for the transfer model at 3% (≈0.16) is only marginally above baseline (≈0.11), and at 10% the baseline median (≈0.43) slightly exceeds that of transfer (≈0.36). These boxplots confirm that pretraining on peptide retention time alone does not confer a reliable advantage for metabolite RT prediction under low-data conditions. (B) Scatterplot of training vs. test performance across all folds and data fractions. Each point represents one cross-validation fold for the single-task pretrained model. Training R^2^ (x-axis) and test R^2^ (y-axis) are plotted; a diagonal line indicates perfect generalisation. Both models have test scores distributed around the same level. Together, panels A and B demonstrate that when the pretraining task is restricted to a single property (RT), the resulting representations do not meaningfully improve metabolite RT prediction beyond a simple baseline, in sharp contrast to the multi-task pretraining that includes RDKit-derived chemical properties.

To determine whether the observed advantage arises specifically from the multi-task pretraining objective (combining RT prediction with RDKit-derived molecular properties), we repeated the experiment using a ChemBERTa model pretrained on a single-task peptide RT prediction (without auxiliary property heads). The same metabolite subsets (3%, 5%, 10%) were used, and performance was compared against the identical baseline (Fig. 4, Table S4).

In sharp contrast to the multi-task model, the single-task pretrained model showed no consistent improvement over the baseline. At 3%, median test R^2^ was 0.156 for transfer vs. 0.108 for baseline; at 5%, 0.313 vs. 0.262; at 10%, 0.364 vs. 0.428. MAE values overlapped substantially across all folds, and neither model systematically outperformed the other. Overall, the single-task pretrained model achieved an approximate parity with the baseline across the entire low-data regime (3–10% of metabolite data). This indicates that pretraining on retention time alone does not confer sufficient chemical knowledge for effective transfer to metabolite RT prediction.

Taken together, these results demonstrate that the auxiliary RDKit property prediction tasks during pretraining equip ChemBERTa with a richer representation of molecular chemistry. This extra chemical information is necessary for efficient transfer learning from peptides to metabolites under extreme data scarcity (3–10% of the metabolite dataset), enabling the model to achieve higher upper-quartile R^2^, lower MAE, and robust gains at 3% and 5% where single-task pretraining fails to improve over a baseline.

### 3.3 Gains from multitask peptide-pretrained model attenuates at 25–100% metabolite data

When more metabolite data became available (25%, 50%, and 100% of the dataset), the benefits of transfer learning diminished, and the baseline model consistently matched or slightly exceeded transfer learning in median and upper-quartile performance (Fig. 3).

At 25% data, the baseline achieved a slightly higher median test R^2^ (0.671, Q3 = 0.671) compared to transfer (0.631, Q3 = 0.642). The baseline’s third quartile (0.671) is also higher than transfer’s third quartile (0.642). Median MAEs were similar (baseline: 74.6, Q3 = 76.6; transfer: 75.1, Q3 = 80.3), but the baseline’s upper quartile for MAE is lower.

At 50% data, median test R^2^ values were nearly identical: transfer 0.729 (Q3 = 0.731), baseline 0.732 (Q3 = 0.738). The baseline’s Q3 (0.738) exceeds transfer’s Q3 (0.731), and the baseline’s median MAE was lower (62.2, Q3 = 64.6) than transfer’s (64.0, Q3 = 68.0).

At 100% of the metabolite data, the baseline slightly outperformed transfer in both median R^2^ (0.803, Q3 = 0.828 vs. transfer 0.790, Q3 = 0.797) and median MAE (51.9, Q3 = 55.1 vs. transfer 53.4, Q3 = 54.3). The third quartile for R^2^ is notably higher for baseline (0.828 vs. 0.797).

Overall, transfer learning from a peptide-pretrained multi-property model provides a clear benefit only under extreme data scarcity (3 to 5%). At 10% the picture is mixed: transfer has a lower median R^2^ but a higher Q3 for R^2^ and better MAE. With 25% or more metabolite data, gains from the multitask peptide-pretrained model attenuate.

**Figure 5:**
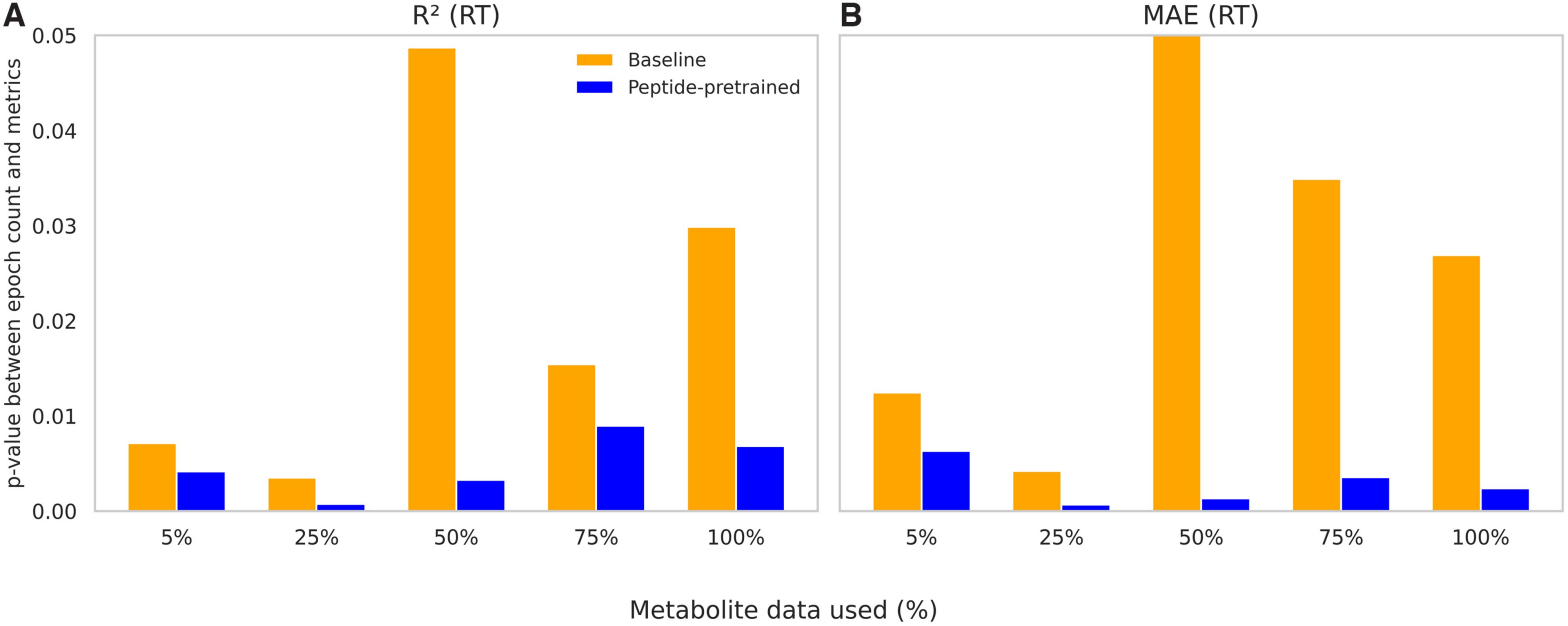
Peptide pre-training accelerates and stabilizes learning dynamics across different data-size scenarios. (A) Bar plots of Pearson correlation *P* value between epoch count and test R^2^, grouped by the percentage of metabolite data used. The peptide-pretrained model (Peptide-pretrained, blue) exhibits consistently lower *P* value across all data fractions, indicating a strong and statistically significant association between training progression and performance improvement. In contrast, the baseline model (Baseline, orange) shows higher and more variable *P* value, particularly at intermediate data sizes, reflecting slower and less stable convergence. (B) Bar plots of Pearson correlation *P* value between epoch count and test MAE. The peptide-pretrained model maintains uniformly low *P* value across all data-size scenarios, demonstrating robust and monotonic error reduction with increasing epochs. The baseline model displays weaker statistical associations at mid-data scenarios, consistent with reduced training stability.

In summary, transfer learning from a multi-property peptide model yields substantial benefits in low-data scenarios and makes training more robust (Fig. 5). This robustness is reflected in the consistently lower *P* value linking epoch count to both test R^2^ and MAE for the peptide-pretrained model, indicating a stable and statistically significant improvement in performance as training progresses. In contrast, the baseline model exhibits weaker and more variable associations, particularly for MAE at intermediate data fractions, consistent with slower convergence and reduced training stability. Together, these results show that peptide-based pre-training not only improves final predictive accuracy but also accelerates learning dynamics and enhances training reliability under data-limited conditions.

## 4 Discussion

We have demonstrated a practical pipeline for small-scale metabolomics prediction: (1) Train a multi-property ChemBERTa model on a large peptide dataset, learning general chemical representations grounded in fundamental descriptors. (2) Transfer this model to a metabolite RT prediction task under data scarcity. This approach significantly outperforms training from scratch, particularly in data-sparse scenarios. At the outset, we observe that the multi-task peptide-pretrained model (ChemBERTa+RDKit) is consistently more robust at low metabolite data scales (3%, 5% and 10%), while reaching performance parity with the baseline from 25% to full data availability. The single-task peptide-pretrained model (ChemBERTa-RT), by contrast, sits at approximate parity with the baseline across the same low-data range, showing that the cross-domain transfer benefit is driven by the auxiliary RDKit objective rather than by peptide pre-training alone. Importantly, no major degradation in performance was observed at larger data scales, indicating an absence of negative transfer from peptide pre-training in this setting. In addition, correlation-based *P* value analyses revealed that the peptide-pretrained model exhibits a more consistent and statistically significant coupling between training epochs and both test R^2^ and MAE across different data-size scenarios, indicating faster convergence and more robust training dynamics compared with the baseline model.

Our findings extend and complement prior work on transfer learning for chromatographic prediction. In proteomics, DeepLC demonstrated that pre-trained peptide RT models can be efficiently adapted to new chromatographic setups and chemically distinct peptide modifications (Bouwmeester *et al.* 2025). In metabolomics, models such as RT-Transformer and hybrid Transformer–LSTM architectures have shown that pre-training on large small-molecule RT datasets improves generalization across chromatographic systems, reporting an R-squared value of 0.89 and 0.91 respectively (Xue *et al.* 2024, Mazraedoost *et al.* 2025). A controlled head-to-head comparison with these methods under identical training budgets and architectures is outside the scope of this proof-of-concept study.

However, these approaches still remain within a single molecular domain. Our work demonstrates effective cross-domain transfer from peptides to metabolites, enabled by multi-property representation learning.

This contrasts with single-task RT models, which may overfit to dataset-specific correlations. The reduced variability and increased stability observed across all metabolite data scales further indicate that multi-property pretraining produces more reliable and reproducible predictors. Even when absolute performance gains diminish at high data availability, transfer learning consistently improves training stability, as evidenced by stable loss behavior, indicating a model that is reliable and capable of maintaining predictive accuracy under varying conditions (Li *et al.* 2025). From a lipidomics and metabolomics perspective, these results are particularly relevant. RT prediction is routinely used to filter candidate annotations and to increase confidence in compound identification in untargeted workflows. Accurate RT prediction under limited data availability could reduce reliance on chemical standards and repeated experimental measurements, thereby accelerating biological interpretation. The success of peptide-to-metabolite transfer further suggests that performance in chromatographic datasets is related to physicochemical principles (Lee *et al.* 2023, Bandini *et al.* 2024), and that it can be learned from data-rich domains and reused in data-poor ones.

More broadly, our work supports the emerging paradigm of foundation models for analytical chemistry, where large, heterogeneous omics datasets are leveraged to build reusable representations that generalize across molecular classes and experimental setups. While this study focused on metabolite RT prediction, our findings provide a clear motivation to investigate whether the proposed strategy is extensible to other metabolomic properties, chromatographic modes, and molecular classes. Future work may explore bidirectional transfer between metabolites and lipids, incorporation of additional auxiliary tasks, or joint modeling across ionization modes and chromatographic conditions (Lee *et al.* 2023, Sibilio *et al.* 2025).

In conclusion, this study establishes multi-task transfer learning from peptides as a robust and scalable solution for metabolite RT prediction under data scarcity. By leveraging large public peptide datasets, our approach addresses a central limitation in lipidomics and metabolomics and provides a generalizable framework for chromatographic property prediction in modern omics research. The quick brown fox jumps over the lazy dog. The quick brown fox jumps over the lazy dog. The quick brown fox jumps over the lazy dog. The quick brown fox jumps over the lazy dog. The quick brown fox jumps over the lazy dog. The quick brown fox jumps over the lazy dog.

## Supplementary Material

btag493_Supplementary_Data

## Data Availability

The code for model definition and training is available at our public GitHub repository (https://github.com/uchealex/chembedding). All data used is publicly available and can be accessed in the METLIN and ProteomicsML databases, as described in the Methods section.

## References

[btag493-B1] Bandini E , Castellano Ontiveros R, Kajtazi A et al Physicochemical modelling of the retention mechanism of temperature-responsive polymeric columns for HPLC through machine learning algorithms. J Cheminform 2024;16:72. 38907264 10.1186/s13321-024-00873-6 PMC11193285

[btag493-B2] Bommasani R , Hudson DA, Adeli E et al On the opportunities and risks of foundation models. arXiv. arXiv: 2108.07258, 2021, preprint: not peer reviewed.

[btag493-B3] Bouthillier X , Delaunay P, Bronzi M et al Accounting for variance in machine learning benchmarks. Proc Mach Learn Sys 2021;3:747–69.

[btag493-B4] Bouwmeester R , Nameni A, Declercq A et al DeepLC introduces transfer learning for accurate LC retention time prediction and adaptation to substantially different modifications and setups. bioRxiv. 2025. 10.1101/2025.06.01.657225 PMC13002937 41667442

[btag493-B5] Bros K , Fabian B, Guba W et al Molecular representation learning for transcriptomics-guided drug discovery. Digit Discov 2023;2:1553–66.

[btag493-B6] Buur LM , Declercq A, Strobl M et al MS2Rescore 3.0 is a modular, flexible, and user-friendly platform to boost peptide identifications, as showcased with MS Amanda 3.0. J Proteome Res 2024;23:3200–7. 38491990 10.1021/acs.jproteome.3c00785

[btag493-B7] Cajka T , Fiehn O. Toward merging untargeted and targeted methods in mass spectrometry-based metabolomics and lipidomics. Anal Chem 2016;88:524–45. 26637011 10.1021/acs.analchem.5b04491

[btag493-B8] Caruana R. Multitask learning. Mach Learn 1997;28:41–75.

[btag493-B9] Chen L , Tan X, Wang D et al Multitask learning with graph neural networks for molecular property prediction. ACS Omega 2021;6:30815–23.

[btag493-B10] Ching T , Himmelstein DS, Beaulieu-Jones BK et al Opportunities and obstacles for deep learning in biology and medicine. J R Soc Interface 2018;15:20170387. 29618526 10.1098/rsif.2017.0387 PMC5938574

[btag493-B11] Chithrananda S , Grand G, Ramsundar B. ChemBERTa: large-scale self-supervised pretraining for molecular property prediction. arXiv. arXiv: 2010.09885, 2020, preprint: not peer reviewed.

[btag493-B12] Devlin J , Chang M-W, Lee K et al BERT: pre-training of deep bidirectional transformers for language understanding. In: *Proceedings of the NAACL-HLT*, pp.4171–86, 2019.

[btag493-B13] Domingo-Almenara X , Guijas C, Billings E et al The METLIN small molecule dataset for machine learning-based retention time prediction. Nat Commun 2019;10:5811. 31862874 10.1038/s41467-019-13680-7 PMC6925099

[btag493-B14] Gan H , Fu L, Zhou R et al WAL-Net: weakly supervised auxiliary task learning network for carotid plaques classification. Eng Appl Artif Intell 2024;137:109144.

[btag493-B15] Greener JG , Kandathil SM, Moffat L et al A guide to machine learning for biologists. Nat Rev Mol Cell Biol 2022;23:40–55. 34518686 10.1038/s41580-021-00407-0

[btag493-B16] Kyle JE. Lipidomics in translational research and the clinical significance of lipid-based biomarkers. Transl Res 2017;189:13–29. 28668521 10.1016/j.trsl.2017.06.006 PMC5659874

[btag493-B17] Landrum G. RDKit: open-source cheminformatics software. 2020. http://www.rdkit.org

[btag493-B18] Lavecchia A. Machine-learning approaches in drug discovery: methods and applications. Drug Discov Today 2015;20:318–31. 25448759 10.1016/j.drudis.2014.10.012

[btag493-B19] Lee IC , Tumanov S, Wong JWH et al Integrative processing of untargeted metabolomic and lipidomic data using MultiABLER. iScience 2023;26:106881. 37260745 10.1016/j.isci.2023.106881 PMC10227420

[btag493-B20] Li J , Lin Y, Gui Z et al Inception–attention–BiLSTM hybrid network: a novel approach for shear wave velocity prediction utilizing well logging. Appl Sci 2025;15:2345.

[btag493-B21] Liu Y , Ott M, Goyal N et al RoBERTa: a robustly optimized BERT pretraining approach. arXiv. arXiv: 1907.11692, 2019, preprint: not peer reviewed.

[btag493-B22] Loshchilov I , Hutter F. Decoupled weight decay regularization. Proc Int Conf Learn Represent, arXiv:1711.05101, 2019, preprint: not peer reviewed.

[btag493-B23] Mazraedoost S , Sedigh Malekroodi H, Žuvela P et al Prediction of chromatographic retention time of a small molecule from SMILES representation using a hybrid transformer-LSTM model. J Chem Inf Model 2025;65:3343–56. 40152775 10.1021/acs.jcim.5c00167

[btag493-B24] Pan SJ , Yang Q. A survey on transfer learning. IEEE Trans Knowl Data Eng 2010;22:1345–59.

[btag493-B25] Paszke A , Gross S, Massa F et al PyTorch: an imperative style, high-performance deep learning library. Adv Neural Inf Process Syst 2019;32:8024–33.

[btag493-B26] Pedregosa F , Varoquaux G, Gramfort A et al Scikit-learn: machine learning in Python. J Mach Learn Res 2011;12:2825–83.

[btag493-B27] Ramsundar B , Eastman P, Walters P et al Deep Learning for the Life Sciences. O’Reilly Media, 2019.

[btag493-B28] Rehfeldt TG , Gabriels R, Bouwmeester R et al ProteomicsML: an online platform for community-curated datasets and tutorials for machine learning in proteomics. J Proteome Res 2023;22:632–6. 36693629 10.1021/acs.jproteome.2c00629 PMC9903315

[btag493-B29] Rives A , Meier J, Sercu T et al Biological structure and function emerge from scaling unsupervised learning to 250 million protein sequences. Proc Natl Acad Sci USA 2021;118:e2016239118. 33876751 10.1073/pnas.2016239118 PMC8053943

[btag493-B30] Sibilio P , De Smaele E, Paci P, Conte F. Integrating multi-omics data: methods and applications in human complex diseases. Biotechnol Rep (Amst) 2025;48:e00938. 41332478 10.1016/j.btre.2025.e00938 PMC12666689

[btag493-B31] Stanstrup J , Neumann S, Vrhovšek U. PredRet: prediction of retention time by direct mapping between multiple chromatographic systems. Anal Chem 2015;87:9421–8. 26289378 10.1021/acs.analchem.5b02287

[btag493-B32] Sterling T , Irwin JJ. ZINC 15 – ligand discovery for everyone. J Chem Inf Model 2015;55:2324–37. 26479676 10.1021/acs.jcim.5b00559 PMC4658288

[btag493-B33] Wang S , Guo Y, Wang Y et al SMILES-BERT: large scale unsupervised pre-training for molecular property prediction. In: *Proceedings of the 10th ACM International Conference on Bioinformatics, Computational Biology and Health Informatics*, pp.429–36, 2019.

[btag493-B34] Wang Y , Xiong J, Xiao F et al LogD7.4 prediction enhanced by transferring knowledge from chromatographic retention time, microscopic pKa and logP. J Cheminform 2023;15:76. 37670374 10.1186/s13321-023-00754-4 PMC10478446

[btag493-B35] Weiss K , Khoshgoftaar TM, Wang D. A survey of transfer learning. J Big Data 2016;3:9.

[btag493-B36] Wolf T , Debut L, Sanh V et al Transformers: state-of-the-art natural language processing. In: *Proceedings of the Conference on Empirical Methods in Natural Language Processing*, pp.38–45, 2020.

[btag493-B37] Wishart DS. Metabolomics for investigating physiological and pathophysiological processes. Physiol Rev 2019;99:1819–75. 31434538 10.1152/physrev.00035.2018

[btag493-B38] Xu Y , Ma J, Liaw A et al Demystifying multitask deep neural networks for quantitative structure–activity relationships. J Chem Inf Model 2017;57:2490–504. 28872869 10.1021/acs.jcim.7b00087

[btag493-B39] Xu Z , Wang X, Wu Z et al Molecular contrastive learning with chemical element knowledge graph. Proc AAAI Conf Artif Intell 2022;36:3968–76.

[btag493-B40] Xue J , Wang B, Ji H et al RT-Transformer: retention time prediction for metabolite annotation to assist in metabolite identification. Bioinformatics 2024;40:btae084. 38402516 10.1093/bioinformatics/btae084 PMC10914443

[btag493-B41] Yang K , Swanson K, Jin W et al Analyzing learned molecular representations for property prediction. J Chem Inf Model 2019;59:3370–88. 31361484 10.1021/acs.jcim.9b00237 PMC6727618

[btag493-B42] Yosinski J , Clune J, Bengio Y et al How transferable are features in deep neural networks? Adv Neural Inf Process Syst 2014;27.

[btag493-B43] Zeng WF , Zhou XX, Willems S et al AlphaPeptDeep: a modular deep learning framework to predict peptide properties for proteomics. Nat Commun 2022;13:7238. 36433986 10.1038/s41467-022-34904-3 PMC9700817

[btag493-B44] Zhang Y , Yang Q. A survey on multi-task learning. IEEE Trans Knowl Data Eng 2022;34:5586–609. 10.1109/tkde.2020.3045924 PMC10619966 37915376

[btag493-B45] Zhuang F , Qi Z, Duan K et al A comprehensive survey on transfer learning. Proc IEEE 2021;109:43–76.

[btag493-B46] Zolg DP , Wilhelm M, Schnatbaum K et al Building ProteomeTools based on a complete synthetic human proteome. Nat Methods 2017;14:259–62. 28135259 10.1038/nmeth.4153 PMC5868332

[btag493-B47] Zvyagin M , Brace A, Hippe K et al GenSLMs: genome-scale language models reveal SARS-CoV-2 evolutionary dynamics. bioRxiv 2022.

