# Supplementary figures and images for: Cross-domain transfer learning from peptides to metabolites using a multi-property fine-tuned LLM
Source: Bioinformatics. 2026 Jul 3;42(7):btag493. doi: 10.1093/bioinformatics/btag493 (PMC13375263; doi:10.1093/bioinformatics/btag493)

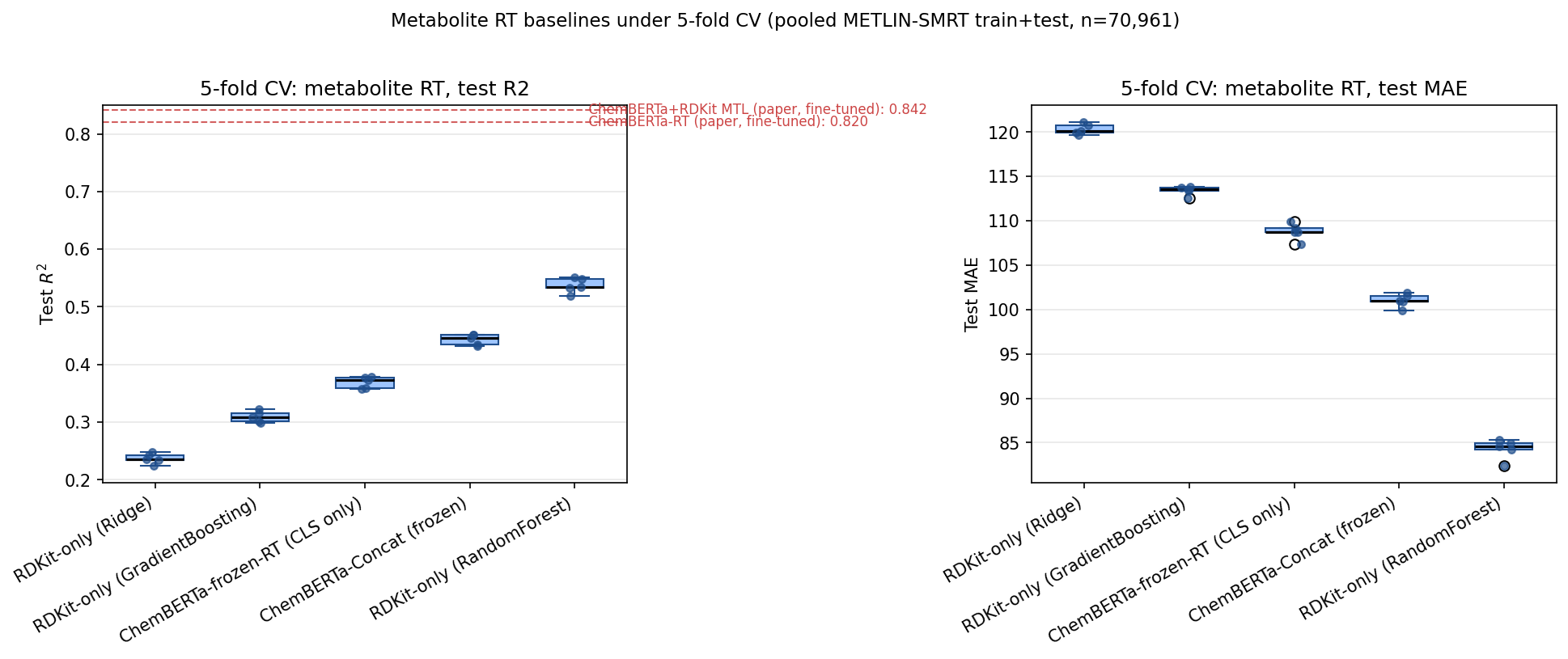

Supplement: btag493_Supplementary_Data [file btag493_supplementary_data.zip › Supplementary_figure_S1_lipid_cv_boxplots.png]
